# Spotlight on the Effect of Workplace Ostracism on Creativity: A Social Cognitive Perspective

**DOI:** 10.3389/fpsyg.2019.01215

**Published:** 2019-05-29

**Authors:** Ming Tu, Zhihui Cheng, Wenxing Liu

**Affiliations:** ^1^College of Economics and Management, Huazhong Agricultural University, Wuhan, China; ^2^School of Business Administration, Hubei University of Economics, Wuhan, China; ^3^School of Business Administration, Zhongnan University of Economics and Law, Wuhan, China

**Keywords:** workplace ostracism, creativity, creative self-efficacy (CSE), collectivism orientation, organization psychology

## Abstract

Drawing on social cognitive theory, we propose a moderated-mediation model to examine when and why workplace ostracism impairs employee creativity in China. We collected 195 valid questionnaires with a two-wave employee-supervisor dyadic research design from one large Chinese company. Results indicate workplace ostracism affects employee creativity negatively. Moreover, the negative effect of workplace ostracism on employee creativity is not only mediated by employee creative self-efficacy (CSE), but the mediation effects of employee CSE are also found to be stronger when employee collectivism orientation is high. Finally, the theoretical and practical implications of these findings are discussed.

## Introduction

With the pressure of growth sustainability and challenges of globalization, contemporary organizations become increasingly reliant on employee creativity to produce novel and useful ideas on products, services, procedures, or practices to ensure longevity and competitiveness (Shalley et al., 2009; Liu et al., 2012). Thus, delving into how employee creativity can be fostered is crucial for both management scholars and practitioners (Shalley et al., 2004). Although a considerable amount of research has explored the antecedents of creativity (see Zhou and Hoever, 2014, for a review), most focused mainly on the positive factors within individuals and organizational context. Surprisingly, researchers appear to have neglected the potential effects of negative factors on employee creativity (Choi et al., 2009). As Baumeister et al. (2001) noted, compared with positive factors within the context, individuals are more sensitive to negative factors, which are influential to their attitudes and behaviors. Given the prevalence and far-reaching effects of workplace ostracism (Williams, 2007), the present study aims to examine the association of workplace ostracism with employee creativity as well as the underlying mechanism and boundary conditions.

Workplace ostracism is defined as “the extent to which an individual perceives that he or she is ignored or excluded at work” (Ferris et al., 2008, p. 1348). As a ubiquitous social phenomenon within organizations, many employees have reported the experience of being ostracized or ostracizing others (Mao et al., 2018). Previous research has indicated that workplace ostracism might undermine an employee’s sense of belonging, control, self-esteem, and meaningfulness (Zadro et al., 2004; Ferris et al., 2015; Wu et al., 2016; Peng and Zeng, 2017), decrease prosocial behaviors (Twenge et al., 2007), and increase counterproductive work behaviors (Hitlan and Noel, 2009; Peng and Zeng, 2017). Nevertheless, few studies have focused directly on the association between workplace ostracism and employee outcomes (Robinson et al., 2013; Wu et al., 2016). Moreover, much less is known on the effects of workplace ostracism on employee creativity (Kwan et al., 2018). Therefore, the present research aims to integrate and extend research on workplace ostracism by examining why and when workplace ostracism undermines employee creativity.

The present study seeks to contribute to extant literature in the following aspects. First, we contribute to a burgeoning stream of research on the outcomes of workplace ostracism by examining its potential negative effect on employee creativity. Second, we provide an explanation for why workplace ostracism hinders employee creativity by identifying creative self-efficacy (CSE) as an important underlying mechanism. Given its negative nature, workplace ostracism is assumed to decrease employees’ CSE, which in turn hampers their creativity. Finally, we draw on social cognitive theory (Bandura, 2001) and propose that collectivism orientation might be an important contingency for the link of workplace ostracism to employee creativity. Collectivism orientation refers to the degree to which individuals base their identities on group membership (Hofstede, 1984, 2001). Compared with individualists, collectivists view themselves as more interdependent with their groups (Triandis, 1995). It is posited that employees with high collectivism orientation tend to be more sensitive to workplace ostracism, which might accentuate the negative relationship between workplace ostracism and employee creativity. In terms of practical implications, we provide evidence for the need for management to pay more attention to the negative effects of workplace ostracism and key issues corresponding to such negative effects.

### Theory and Hypotheses

#### Workplace Ostracism and Creativity

Workplace ostracism reflects one’s perception of ignorance and exclusion of others in the workplace (Ferris et al., 2008). We argue that workplace ostracism undermines employee creativity for several reasons. First, ostracism brings about an unpleasant experience to employees, which might affect their work efforts further (Williams, 2001, 2007). For example, some scholars have demonstrated that workplace ostracism results in employees’ unwillingness to dedicate extra efforts to benefit their organizations (e.g., Twenge et al., 2007; Balliet and Ferris, 2013). As such, employees who experience workplace ostracism might not offer creative ideas and solutions to improve their organizational effectiveness.

Second, workplace ostracism might increase employees’ perception that new ideas are disparaged or unwelcome. As posited by Amabile et al. (2002), generating creative ideas requires a sense of control in accessing relevant resources, whereas workplace ostracism reduces an individual’s sense of external control and flexibility in enacting novel ideas, which are crucial to employee creativity. In addition, workplace ostracism will undermine individuals’ access to the resources needed for producing creative ideas. For example, Kwan et al. (2018) demonstrate that ostracism from supervisors can hinder employees’ creativity. Considering the above arguments, we hypothesized that

Hypothesis 1: Workplace ostracism is negatively associated with employee creativity.

#### Workplace Ostracism and Employee’s Creativity via CSE

Creative self-efficacy represents one’s belief in one’s ability to produce creative outcomes (Tierney and Farmer, 2002, 2011). Drawing on the social cognitive perspective (Bandura, 2001), we posit that workplace ostracism might hamper employees’ creativity by decreasing his/her CSE. First, employees’ CSE is regarded as an important antecedent of employee creativity, because employees might not generate creative ideas or actions without sufficient CSE (Tierney and Farmer, 2002). However, one’s CSE is likely to be impaired by job characteristics or organizational contexts. Based on Williams’s (2001) model of ostracism, workplace ostracism can pose a threat to employees’ sense of belonging and self-esteem. Individuals have a fundamental need to have positive and stable interactions with in-group members. The absence of these interactions will decrease an individual’s self-esteem and self-worth (Ferris et al., 2015; Wu et al., 2016), which is the main determinant of self-efficacy (Bandura, 2002). Therefore, we assume that workplace ostracism will decrease employees’ CSE.

Second, employees must exert extra efforts to try new methods and procedures to maintain creativity, which requires orientation to master goals and the involvement of creativity, both of which are correlated to CSE. Prior research has indicated that employees with high CSE are oriented toward goal mastery (Beghetto, 2006) and creative work (Carmeli and Schaubroeck, 2007). Many empirical studies have found that CSE has a positive effect on creativity (Tierney and Farmer, 2002, 2011; Gong et al., 2009). Moreover, recent meta-analytic research has demonstrated CSE is not only associated positively with creativity but is also an important underlying mechanism to understand creativity (Liu et al., 2016). Therefore, we predict the following:

Hypothesis 2: Workplace ostracism is negatively associated with CSE.
Hypothesis 3: Workplace ostracism has a negative indirect effect on employee creativity via CSE.

#### Moderating Role of Collectivism

Collectivism refers to the degree to which individuals base their identities on group membership (Hofstede, 1984, 2001). In the present research, we focus mainly on individual level collectivism, which has been found to affect an individual’s conceptions, attitudes, behaviors, and its relationships with other variables (Triandis and Gelfand, 1998; Oyserman et al., 2002).

According to the social cognitive theory (Bandura, 2001), collectivism is likely to affect an individual’s attitudinal response to workplace ostracism. Thus, we posit that collectivism might act as a contingent factor between workplace ostracism and employee’s CSE. First, because of the fundamental role that values play in shaping individuals’ goals and behaviors (Shin and Zhou, 2003), differences in values may affect substantially the way individuals respond to workplace ostracism. Specifically, compared with low collectivism-oriented individuals, high collectivism-oriented individuals value the interpersonal connection with others and put the interests of their identified group first. Second, several studies have indicated that individuals with high collectivism orientation are very sensitive to interpersonal information (Cross et al., 2000; Powell et al., 2009; Hofman and Newman, 2014). Experiencing ostracism, individuals are likely to exhibit more intense psychological and behavioral responses, such as a decreasing sense of belonging to their group. According to the belongingness theory (Baumeister and Leary, 1995), individuals need only a certain level of social connectedness for belongingness to be fulfilled. Lacking these fundamental needs will decrease employees’ CSE. Therefore, when employees with high collectivism orientation are ostracized, their CSE will be more greatly impaired. As such, we hypothesize the following:

Hypothesis 4: Collectivism orientation moderates the relationship between workplace ostracism and employee CSE, such that the relationship is stronger for those employees with a high rather than low collectivism orientation.

In line with the moderated-mediation logic of Edwards and Lambert (2007), we combine the above hypotheses and propose that

Hypothesis 5: Collectivism orientation moderates the indirect effect of CSE on the relationship between workplace ostracism on employee creativity such that the indirect effect will be stronger for those employees with a high rather than low collectivism orientation.

## Materials and Methods

### Research Setting, Sample, and Procedures

The present study was carried out in accordance with the recommendations of the Ethics Committee of School of Economics and Management, Huazhong Agricultural University with written informed consent from all subjects. All subjects gave written informed consent in accordance with the Helsinki Declaration. The protocol was approved by the Ethics Committee of School of Economics and Management, Huazhong Agricultural University. Employees and their immediate supervisors from one large telecommunication company in China participated in our investigation. We conducted semi-structured interviews with six managers from the marketing, human resources management, financial, production, administrative, and supply departments to validate the survey items. Furthermore, to guarantee the respondents’ anonymity, we obtained the name lists of the supervisors and employees from the HR and gave every group a code number, then every member in the group a sub-number. For example, group one has one supervisor and four employees. We coded the supervisor as 10, and the four employees as 11, 12, 13, and 14, respectively. The questionnaires had space for their code numbers. One of the co-authors visited the company and conducted the survey during regular working days. On our first visit, with the help of HR manager, we delivered the questionnaires along with envelopes to the focal employees, which included their demographic information and their perceptions of workplace ostracism, CSE, and collectivism. To avoid matching error issues, we sorted employees from the same groups or departments in the same envelope. On our next visit (about 1 month later), with the help of the HR manager, their immediate supervisors rated the employees’ creativity for the subsequent time after the last survey. Finally, the HR manager collected the supervisors’ questionnaires along with their subordinates, sealed them into envelopes, and returned them to our investigator representative. In summary, we distributed questionnaires to 230 employees and 50 supervisors separately with the help of the HR manager. However, we ultimately received 195 completed and usable questionnaires in pairs, representing an appropriate 84.8% response rate.

Among the respondents, 104 were female (53.3%). In terms of educational attainment, 53 employees (27.2%) had a high school diploma or lower degree, 63 employees (32.3%) had an institute of technology or lower degree, 62 employees (31.8%) had a bachelor’s degree, and 17 employees (8.7%) had a master’s degree or higher. The average length of employment in current positions was 3.57 years (*SD* = 4.13). The average age of employees was 28.59 (*SD* = 4.99).

### Measures

Chinese versions of all measures were created following Brislin’s (1986) translation-back-translation procedure. Unless otherwise noted, all items used in the present study were measured by seven Likert-type scales anchored from 1 (strongly disagree) to 7 (“strongly disagree).

#### Workplace Ostracism

The employees were asked to report their perception of workplace ostracism using a 10-item scale adapted from Ferris et al. (2008). Sample items included “Others ignored you at work” and “You noticed others would not look at you at work” (Cronbach’ α = 0.94).

#### Creativity

The immediate supervisors were asked to use a four-item creativity scale developed in the Chinese context by Farmer et al. (2003) to access their subordinates’ creativity. A sample item was “This employee generates ground-breaking ideas related to the field.” In the present study, the Cronbach’ α for this scale was 0.91.

#### Creative Self-Efficacy

Creative self-efficacy was measured using the three-item scale developed by Tierney and Farmer (2002). One sample item was “I have confidence in my ability to solve problems creatively” (Cronbach’ α = 0.83).

#### Collectivism

In the present study, we adopted the five-item collectivism scale derived from Triandis and Gelfand (1998). A sample item was “I like to live close to my good friends.” In this study, the Cronbach’ α for this scale was 0.91.

#### Control Variables

Accounting for the heterogeneity of the sample, we controlled four demographic variables (i.e., gender, age, education, and tenure), because these variables have been found to be related significantly to creativity (Shalley et al., 2004; Kwan et al., 2018). Age was measured in years. Gender was measured as a dichotomous variable coded as “0” for male and “1” for female. Education was measured on a four-point scale (one = high school, two = institute of technology, three = bachelor’s, four = master’s). Position tenure was measured as the number of years that an employee had been in the current position.

## Results

### Preliminary Analysis

Given that the workplace ostracism, CSE, and collectivism ratings were reported by employees with common seven Likert-type scale formats. We first tried to preclude the common method variance (CMV). Specifically, we adopted Harman’s single-factor statistical remedy recommended by Podsakoff et al. (2003). Using principal component analysis, output revealed four distinct factors accounting for 72.41% of the total variance. The first unrotated factor captured only 35.88% of the variance in data, indicating our results did not meet the two underlying assumptions of CMV issue. We also employed the remedy conducted by Liang et al. (2007) to detect CMV. Specifically, we included in the PLS model a common method factor whose indicators included all the principal constructs’ indicators and calculated each indicator’s variances explained substantively by the principal construct and by the method. As is shown in Appendix Table A1, the result demonstrates the average explained substantively the variance of the indicator is 0.646, while the average method-based variance is 0.32. The ratio of substantive variance to method variance is about 2:1. Combining the two methods, we contend that CMV might not be a serious issue in this study.

**Table 1:** Confirmatory factor analyses.

Model	χ^2^	*df*	Δχ^2^*(Δdf)*	CFI	TLI	RMSEA
(1) Hypothesized four-factor model	164.76	59	–	0.95	0.94	0.09
(2) Three-factor model: (CSE + Creativity)	414.00	62	249.24^∗∗∗^ (3)	0.84	0.80	0.17
(3) Two-factor model: (Ostracism + Collectivism, CSE + Creativity)	962.05	64	797.29^∗∗∗^ (5)	0.60	0.52	0.26
(4) Single-factor model	1648.43	65	1483.67^∗∗∗^ (6)	0.31	0.17	0.35

*CSE, creative self-efficacy, “+” means combined. All alternative models were compared with the hypothesized four-factor model. CFI, comparative fit index; TLI, Tucker-Lewis index; RMSEA, root mean squared error of approximation. ^∗∗∗^p < 0.001.*

Then, we conducted a confirmatory factor analysis (CFA) in M*plus* 7.4 statistical software developed by Muthén and Muthén (2015) to check the discriminant validity of our focal constructs. We adopted item parceling techniques for the parsimony of the measurement model (Williams and Anderson, 1994). Specifically, following Little et al. (2002) recommendation, we parceled workplace ostracism and collectivism into three items. As depicted in Table 1, the proposed four factors (i.e., workplace ostracism, CSE, collectivism, and creativity) reached an acceptable fit level (χ^2^ = 164.76, *df* = 59, CFI = 0.95, TLI = 0.94, RMSEA = 0.09). Moreover, the fit index indicates the hypothesized four-factor model excelled over the three-factor model (i.e., combining CSE and creativity into one single factor: χ^2^= 414.00, *df* = 62, CFI = 0.84, TLI = 0.80, RMSEA = 0.17;Δχ^2^ = 249.24, *df* = 3, *p* < 0.001) and the baseline single factor model (i.e., loading all items as one single factor:χ^2^= 1648.43, *df* = 65, CFI = 0.31, TLI = 0.17, RMSEA = 0.35; Δχ^2^= 1483.67 *df* = 6, *p* < 0.001). Thus, the CFA results indicate the discriminant validity our constructs is well-established.

**Table 2:** Means, standard deviations, and correlations among study variables.

Variable	*Mean*	*SD*	1	2	3	4	5	6	7	8
(1) Gender	0.53	0.50								
(2) Age	28.59	4.99	0.01							
(3) Education	2.22	0.95	−0.12	−0.02						
(4) Tenure	3.58	4.01	0.01	0.64^∗∗^	−0.01					
(5) Workplace ostracism	4.97	0.94	−0.06	0.07	0.05	0.06	**(0.94)**			
(6) Creative self-efficacy	2.76	1.04	−0.01	−00.01	−0.11	−0.05	−0.37^∗∗^	**(0.83)**		
(7) Collectivism	4.01	1.35	−0.21^∗∗^	0.06	−0.19^∗∗^	0.17^∗^	0.15^∗^	−0.26^∗∗^	**(0.91)**	
(8) Creativity	3.80	1.10	0.00	−0.06	0.07	−0.10	−0.29^∗∗^	0.33^∗∗^	−0.22^∗∗^	**(0.91)**

*(1) N = 195. Gender is coded 0 = male, 1 = female; (2) ^∗^p < 0.05, ^∗∗^p < 0.01; (3) Bracketed bold values on the diagonal are the Cronbach’s alpha value of each scale.*

#### Main Analyses

Although our data were collected from 195 employees of 50 teams, we did not adopt a multilevel structural equation modeling because of the small variance between groups. Because we calculated the one-way ANOVAs for all key constructs in SPSS 23.0, and results indicated a non-significant difference between groups for workplace ostracism (*F* = 0.817, *p* = 0.790), collectivism (*F* = 0.892, *p* < 0.671), Creativity (*F* = 0.964, *p* < 0.547), except creative self-efficacy reached significant level (*F* = 1.610, *p* = 0.016). Then, we calculated the intra-class correlations (ICCs) in Mplus 7.4, the ICCs for the workplace ostracism creativity, CSE, and collectivism were 0.003, 0.033, 0.127, and 0.020, respectively. Therefore, we adopted a single-level analysis of the whole model. Table 2 presents the means, standard deviations, and correlations among all variables. Results showed workplace ostracism was correlated significantly and negatively with both creativity (*r* = −0.29, *p* < 0.01) and CSE (*r* = −0.37, *p* < 0.01). Meanwhile, CSE was correlated significantly and positively with creativity (*r* = 0.33, *p* < 0.01).

**FIGURE 1:**
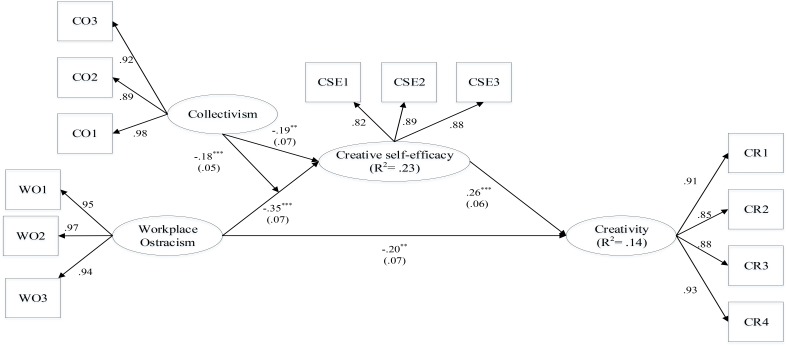
Structural model results. Path coefficients: bootstrapping = 10,000, ^∗∗^*p* < 0.01, ^∗∗∗^*p* < 0.001. Bracketed values are standard errors.

We adopted structural equation modeling to test the hypotheses via Smart-pls 3.0 (Ringle et al., 2015) and the results are shown in Figure 1. Hypothesis 1 predicted that workplace ostracism is related negatively to creativity. The result indicates that the total effect between workplace ostracism and creativity is −0.29 (*SE* = 0.07, *p* < 0.001), and hence, Hypothesis 1 is supported. Hypothesis 2 predicted that workplace ostracism was negatively related to employee CSE. The result indicates that workplace ostracism was related negatively to employee CSE (β = −0.35, *SE* = 0.07, *p* < 0.001), and thus, Hypothesis 2 is supported.

Hypothesis 3 proposed that CSE mediated the relationship between workplace ostracism and creativity. Figure 1 shows the path coefficients between CSE and workplace ostracism (β = −0.35, *SE* = 0.07, *p* < 0.001) and creativity (β = 0.26, *SE* = 0.06, *p* < 0.001) are significant, providing initial support to Hypothesis 3. In addition, we adopted the resampling methods to test the robustness of the indirect effects (Mackinnon et al., 2004). Based on 10,000 Monte Carlo replications, the bootstrapping results revealed the indirect relationship between workplace ostracism on employee creativity via CSE is significant (*Indirect Effect* = −0.09, 95% CI = [−0.153, −0.038]). Therefore, Hypothesis 3 is supported.

**FIGURE 2:**
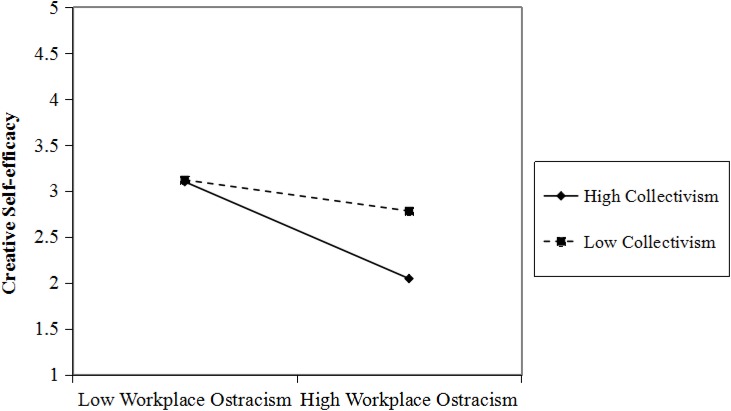
Interactive effect of collectivism and workplace ostracism on employee’s creative self-efficacy.

Hypothesis 4 proposed that collectivism moderated the relationship between workplace ostracism and CSE. Figure 1 shows the interaction term of workplace ostracism and collectivism is related negatively to employee CSE (β = −0.18, *SE* = 0.05, *p* < 0.001). Then, following the recommendation of Aiken and West (1991), we depicted the effects of the two-way interaction proposed in Hypothesis 4 (Figure 2) and conducted the simple slope tests. Results indicated the negative relationship between workplace ostracism and CSE was stronger when employees’ collectivism was high (β = −0.59, *SE* = 0.09, *p* < 0.001) than when it was low (β = −0.11, *SE* = 0.09, *p* = 0.24). Thus, Hypothesis 4 is supported.

Hypothesis 5 predicted that the indirect effect of workplace ostracism on employee creativity via CSE would be stronger when an employee’s collectivism is high. We tested the moderated mediation effect in accordance with the analytical procedures recommended by Edwards and Lambert (2007). The indirect effect of CSE is more significant when employees’ collectivism was high (*Estimate* = −0.15, 95% CI = [−0.242, −0.077]) than when it is low (*Estimate* = −0.03, 95% CI = [−0.082, 0.018]). Moreover, the difference in magnitude of the two indirect relationship is significant (Difference = −0.12, 95% CI = [−0.225, −0.031]). Thus, Hypothesis 5 is supported.

## Discussion

The present research integrates ostracism and creativity literature by demonstrating when and why workplace ostracism relates negatively to employee creativity in the Chinese context. Our hypothesized model is supported by 195 dyadic data from one large telecommunication company. Overall, we found that workplace ostracism is related negatively to employee creativity. Moreover, this negative relationship is mediated by CSE. In addition, employee collectivism accentuates not only the negative relationship between workplace ostracism and CSE but also reinforces the indirect effect of CSE. Specifically, the indirect effect of CSE between workplace ostracism and creativity was significant only when employees’ collectivism was higher.

### Theoretical Contributions

The findings of this research contribute to extant literature in the following manner. First, the present study is one of the few studies to probe into the association between workplace ostracism and employee creativity. As Hitlan et al. (2006) noted, future scholars need to extend the outcomes of workplace ostracism. To some extent, this research advances understanding on the negative impact of workplace ostracism on employee creativity. Prior research regarding the link between organizational climate and creativity focused mainly on identifying a positive organizational climate that facilitated employees’ creativity, such as an innovative climate, team psychological safety, and a supportive atmosphere. Consequently, the influence of negative factors in organizational climates (i.e., workplace ostracism) on creativity has been largely neglected. Consistent with the latest research by Kwan et al. (2018), we also found workplace ostracism can inhibit employee’s creativity. In summary, bridging research on workplace ostracism and creativity advances our understanding on why workplace ostracism is so detrimental to both employees and organizations.

Second, in response to Robinson et al.’s (2013) call to probe into the relationships between workplace ostracism with psychological and behavioral outcomes, this study provides a comprehensive model to understand the underlying mechanism of the negative relationship between workplace ostracism with employee creativity by examining the mediating role of CSE. Although prior literature has provided compelling support for the notion that negative events were associated negatively with employee creativity (Zhou and Shalley, 2003), few studies have explored directly the mechanism through which workplace ostracism, a ubiquitous social phenomenon within organizations, affects employee creativity. Our findings show workplace ostracism is related negatively to CSE, which in turn relate to employee creativity.

Finally, the present study contributes to the boundary condition research on workplace ostracism and consequences by accounting for the moderating role of collectivism. As to which types of employees are more sensitive to workplace ostracism, our findings demonstrated that the detrimental effect of workplace ostracism on employee’s creativity is more pronounced when employees are characterized by high collectivism orientation.

### Practical Implications

The present research also offers managerial insights to stimulate and protect employees’ creativity. First, our findings demonstrate that workplace ostracism is detrimental to employee creativity. Thus, management should focus more attention to the occurrences of ostracism phenomena at work and provide appropriate intervention to control the negative effects of workplace ostracism.

Second, our results show that workplace ostracism hinders employee’s CSE. Because CSE is essential for employees to generate creative ideas (Liu et al., 2016), employees who lack CSE are unlikely to produce creative outcomes. Thus, management should train employees on how to promote their CSE in relation to the improvement of organizations.

Finally, our research identified that the negative effect of workplace ostracism on employee creativity was contingent on employee’s collectivism. For management, employees with high collectivism orientation deserve more attention because employees with high collectivism are more sensitive to workplace ostracism and their CSE and creativity are impaired more easily.

### Limitations and Future Research

The present study is far from perfect and has certain several limitations. First, the cross-sectional research design precluded the causal inference of the effect of workplace ostracism on employee creativity. In other words, our findings were consistent with our theoretical reasoning. The cross-sectional design did not allow for alternative explanations to be ruled out completely. Hence, future research can adopt a longitudinal or experimental design to strengthen the robustness of our findings.

Second, the study has two drawbacks in terms of research design. The present study cannot alleviate the common method variance completely. Although we conducted *post hoc* remedies to detect the common method variable via two statistical methods (Podsakoff et al., 2012), the CMV cannot be ruled out. Thus, future research should employ a more rigid research design. Moreover, we only collected data from only one company in the Chinese telecommunications industry, which undermines the generalization of our findings. Nevertheless, conducting this study in a single organization had the advantage of controlling for potential organization-level confounding variables. However, future research within multiple organizational settings could increase the generalizability of the findings to other types of employees and organizations. Moreover, data analysis result indicated some group level effect (e.g., ICC for CSE is 0.127). Researchers can probe into the group level effect of workplace ostraism within organizations in future research.

Finally, we only examined one psychological mechanism between workplace ostracism and creativity. However, underlying mechanism research is still insufficient. From a self-concept perspective, workplace ostracism might impair one’s self-esteem within the organization or from a resource conservation perspective, workplace ostracism might bring about exhaustion to employees when they deal with such social obstacles. Therefore, future research could advance understanding of the relationship between workplace ostracism and creativity by examining additional mediating effects (e.g., organizational-based self-esteem).

## Conclusion

In the present study, we draw on the social cognitive perspective to explain the cognitive and behavioral consequences of workplace ostracism. Our findings demonstrate that workplace ostracism can hamper employee’s CSE, which in turn decreases employee creativity, especially with high collectivism of focal employees. Although literature regarding the relationship between workplace ostracism and employee creativity is still nascent, we hope to advance understanding of such issue within organizations.

## Author Contributions

MT and WL adopted the study and wrote the draft paper. WL collected the data. ZC systematically re-analyzed the data and rewrote the manuscript, and communicated with the reviewers. MT and ZC provided the fund support.

## Conflict of Interest Statement

The authors declare that the research was conducted in the absence of any commercial or financial relationships that could be construed as a potential conflict of interest.

## Appendix

**Table A1:** Common method bias analysis.

Construct	Indicator	Substantive factor loading(R_1_)	R_1_^2^	Method factor loading(R_2_)	R_2_^2^
Workplace ostracism	WO1	0.665	0.442	0.663	0.440
	WO2	0.656	0.430	0.650	0.423
	WO3	0.730	0.533	0.733	0.537
	WO4	0.919	0.845	0.915	0.837
	WO5	0.732	0.536	0.738	0.545
	WO6	0.726	0.527	0.730	0.533
	WO7	0.689	0.475	0.703	0.494
	WO8	0.890	0.792	0.887	0.787
	WO9	0.896	0.803	0.901	0.812
	WO10	0.966	0.933	0.960	0.922
Creative self-efficacy	CSE1	0.708	0.501	−0.318	0.101
	CSE2	0.833	0.694	−0.339	0.115
	CSE3	0.829	0.687	−0.336	0.113
Collectivism orientation	CO1	0.954	0.910	0.065	0.004
	CO2	0.677	0.458	0.027	0.001
	CO3	0.632	0.399	0.169	0.029
	CO4	0.972	0.945	0.076	0.006
	CO5	0.623	0.388	0.169	0.029
Creativity	CR1	0.870	0.757	0.317	0.100
	CR2	0.754	0.569	0.239	0.057
	CR3	0.834	0.696	0.234	0.055
	CR4	0.946	0.895	0.313	0.098
Average		0.796	0.646	0.386	0.320
